# Is a Forest Fire a Natural Disaster? Investigating the Fire Tolerance of Various Tree Species—An Educational Module

**DOI:** 10.3390/biomimetics9020114

**Published:** 2024-02-15

**Authors:** Olga Speck, Thomas Speck

**Affiliations:** 1 Cluster of Excellence liv MatS @ FIT—Freiburg Center for Interactive Materials and Bioinspired Technologies, University of Freiburg, Georges-Köhler-Allee 105, 79110 Freiburg, Germany; thomas.speck@biologie.uni-freiburg.de; 2 Plant Biomechanics Group @ Botanic Garden Freiburg, University of Freiburg, Schänzlestr. 1, 79104 Freiburg, Germany

**Keywords:** educational module, fire tolerance, pyrophytes, tree bark, wildfire

## Abstract

Wildfires are unplanned conflagrations perceived as a threat by humans. However, fires are essential for the survival of fire-adapted plants. On the one hand, wildfires cause major damage worldwide, burning large areas of forests and landscapes, threatening towns and villages, and generating high levels of air pollution. On the other hand, fire-adapted plants (pyrophytes) in the fire landscapes of the Earth are able to survive exposure to heat (e.g., because of their thick bark, which protects their living tissue) and benefit from fire directly (e.g., fire initiates cone opening and seed release) or indirectly (e.g., fewer competing plants of fire-sensitive species remain, seeds germinate in the ash-fertilized soil). We present the experimental set-up and results of a fire experiment on bark samples used as a basis to assess the fire tolerance of various trees. Fire tolerance is defined as the ability of a tree to survive a surface fire (up to 200 °C and 5 min duration). The measure of the fire tolerance for a tree species is the time taken for the vascular cambium under the insulating bark to reach the critical temperature of 60 °C. Within an educational module, we provide worksheets for teachers and students enabling them to analyze the fire tolerance of various tree barks.

## 1. Introduction

Wildfires are unplanned, uncontrolled, and unpredictable fires that can burn large areas of land for days or weeks. Depending on the vegetation that is being burned, wildfires are also known as forest fires, grass fires, peat fires, or bush fires. In recent years, reports and images of devastating wildfires have circulated around the world. In the European Union, 2022 was the second worst wildfire season, which was surpassed only by that of 2017. In 2022, fires were mapped in 26 of the 27 EU countries (all except Luxembourg), burning a total of 8372 km^2^ [1].

In this publication, we provide background information on wildfires (Section 1), fire landscapes of the Earth (Section 2), fire-adapted plants (Section 3), and possible technical applications of fire-resistant tree barks (Section 4) as the basis for an educational module on fire tolerance of trees, which is a characteristic that can be investigated by flaming tests of bark samples (Section 5). The educational module includes four working sheets that are provided in the Supplementary Materials File S1: (1) Information: Fire tolerance of trees, (2) Research: Tasks for independent preparation, (3) Experiment: Flaming test of bark samples, and (4) Solution: Answers to all tasks and discussion of the experimental results.

### 1.1. Wildfires—Advantages and Disadvantages

In the context of worse wildfire events occurring around the world, the question arises as to whether wildfires are a curse or a blessing for nature. The answer is “probably both”. On the one hand, wildfires are an integral part of many of the Earth’s ecosystems [2,3]. They act as an evolutionary pressure that shapes plant traits (e.g., seed and bark traits) and generates fire adaptations [4]. In biology, adaptations are understood as hereditary genetic changes in plant populations over evolutionary time [5]. On the other hand, humans perceive wildfires as a dangerous natural threat. Wildfires have social, environmental, and economic impacts on human society, including the loss of public and private assets and the destruction of critical infrastructure, and they are a significant danger for human health and safety [2]. Schultz et al. [6] present the global wildfire emissions from 1960 to 2000 that may be the dominant factor controlling the interannual variability of the atmospheric composition. According to the World Health Organization (WHO), smoke from a wildfire is a mixture of hazardous pollutants that contaminate the air and can cause and exacerbate human illness. Particulate matter is one of the greatest health hazards in forest fire smoke. The very small particles (PM_2.5_ = 50% of particles with a diameter of 2.5 µm), accompanied by a higher proportion of smaller particles and a lower proportion of larger particles), are particularly associated with various diseases because they can penetrate very deeply into the lungs and cause damage [7].

### 1.2. Occurrence, Severity and Origin of Wildfires

**Figure 1:**
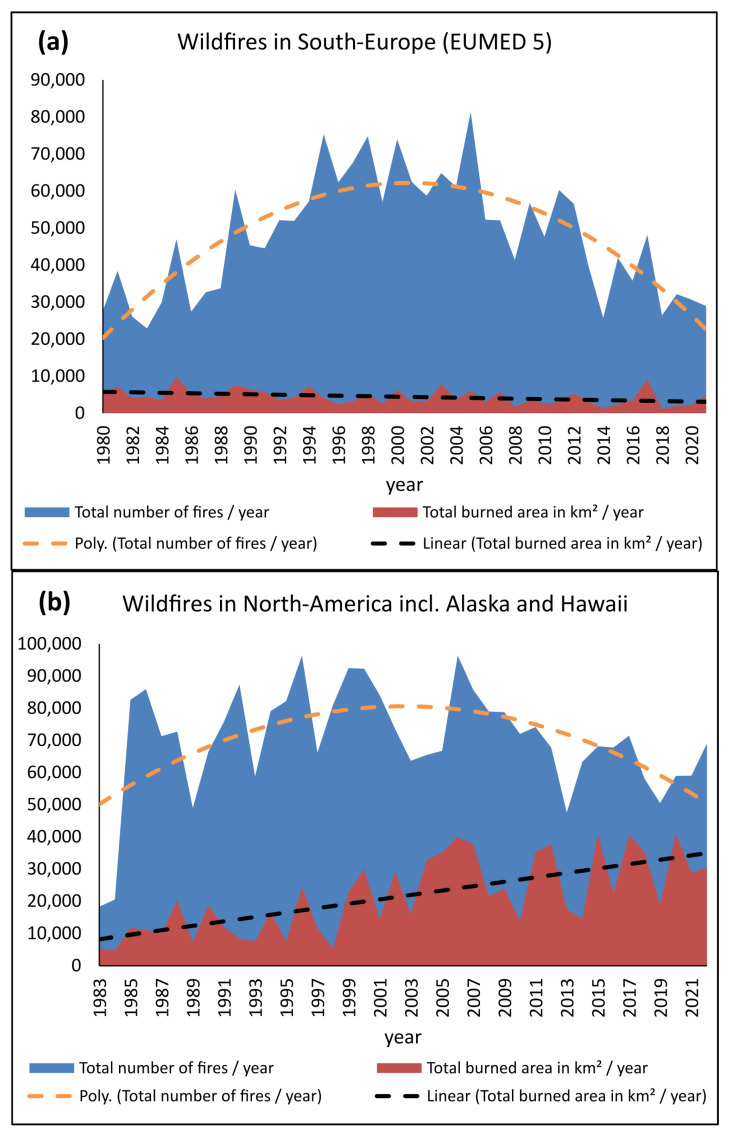
Number of wildfires and burned area (in km^2^) per year in (**a**) European Mediterranean countries (EUMED 5 = Portugal, Spain, southern France, Italy, and Greece) and (**b**) North America including Alaska and Hawaii. Total number of wildfires (blue) with polynomial trend line (orange), total burnt area (red) with linear trend line (black) (data from [8,10]).

Forest fires are monitored worldwide in terms of the annual number of fires, the burned area per year, and the countries or landscapes affected. The figures are freely available on various official websites on the Internet. The analysis of the figures of the European Mediterranean countries (Figure 1a [8]) and North America, including Alaska and Hawaii (Figure 1b [9,10]) show marked fluctuations from one year to the next. The overall trend is an increase in the number of fires during the 1990s, arising partly because of improved recording methods, and a general downward trend since the early 2000s. An increase in area burned can be seen in North America between 1983 and 2022 [10]. In contrast, the annual burned area in the five large Mediterranean countries of Europe show a decrease in the period from the 1980s to the 2021s [8] with peaks in 2017 and 2022 [1,11]. An analysis of wildfires in fire-prone European Mediterranean landscapes indicates that large wildfires are becoming more frequent [12]. Data used are given in Supplementary Materials File S2.

Globally, only 4% of all forest fires have natural causes such as extreme weather events (high temperatures, drought and storms), lightning, or volcanic eruptions, whereas up to 96% of forest fires are caused by humans through unauthorized or careless activities [13]. In North America, including Alaska and Hawaii, an average of 87% of wildfires were caused by humans, 13% being attributable to lightning, from 2001 to 2022 [14,15]. In the 1980s–2010s period, only 5% of forest fires were the result of natural events such as lightning strikes, the other 95% being attributed to human carelessness or arson. Nowadays, more than 90% of the wildfires in the Mediterranean region are human-caused [16]. In recent years, various methodologies and indexes have emerged to determine the likelihood of wildfire. Mhawaj et al. [17] present 28 factors from a literature review associated with the ignition of wildland fires and divide them into climatic, topographical, in-situ, historical, and anthropogenic factors.

### 1.3. Wildfire Types

**Figure 2:**
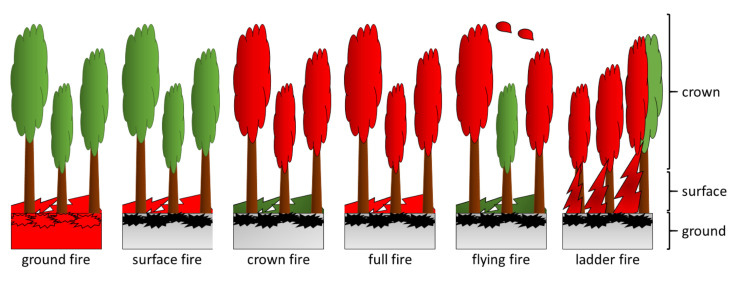
The type of forest fire depends on whether the ground with the roots, the surface with the undergrowth, and/or the crown are burning. In a flying fire, pieces of vegetation are carried by the wind far beyond the fire front. A ladder fire arises when low-growing burning vegetation carries the fire to taller vegetation. Burning structures are shown in red.

A distinction is made between types of forest fires, as they can have a variety of effects on the vegetation (Figure 2) [18]. Depending on the material burned, various pollutants are released into the air such as particulate matter (PM_2.5_), polycyclic aromatic hydrocarbons (PAHs), and carbon monoxide, which can cause and exacerbate diseases of the lungs, heart, brain/nervous system, skin, gut, kidney, eyes, nose and liver [7]. Among the wildfire types, the crown and flying fires are the most dangerous for human safety because they are fast-moving and high-intensity fires [19]. Ground fires or smoldering fires are mostly flameless and spread slowly, burning through thick organic layers such as raw humus, moss, and peat. The negative impact of these “hot fires” on tree vegetation, which can exceed 500 °C, is particularly dramatic, as the root systems of externally intact trees are killed by smoldering fires. Surface fires spread rapidly over the ground surface, mainly burning easily combustible material such as undergrowth, shrubs, herbs, grasses, and leaf litter. As surface fires are “cold fires” with temperatures of up to 200 °C, damaged trees and shrubs regrow relatively quickly. The rapid passage of surface fires, which often burn for only a few seconds, also has a positive effect on species survival especially in the case of highly fire-adapted tree species with a thick bark. Crown fires rarely occur in isolation but are usually associated with surface fires. When crown fires occur alone, the crowns of the trees burn without the fire spreading to the litter or stem layer. With temperatures above 1000 °C, crown fires destroy the affected trees. These fires are not usually separate events. Ground, surface, and crown fires can occur simultaneously in a forest fire or as a ladder fire that consumes material sequentially between low-level vegetation and tree canopies. A full fire is a combination of surface and crown fires and often results in the complete destruction of a tree stand, as the large amount of combustible material can reach extremely high temperatures of up to 500 °C; these fires frequently burn for a long time. In flying fires, also known as spot fires, burning vegetation such as bark, twigs, leaves, and cones are transported beyond the fire front by the wind and by thermals resulting from the fire to areas where they can ignite new fires. Since sparks and embers can be carried considerable distances, they can remain undetected for some time, and the resulting spot fires pose a serious hazard to the local population and any firefighting personnel.

### 1.4. Aim of the Project

In this article, we present basic information about wildfires, which are an integral part of many of the Earth’s ecosystems but are also perceived by humans as a dangerous natural threat. We focus on fire-adapted plants (pyrophytes) in the world’s fire landscapes that are able to survive exposure to high temperatures and benefit directly or indirectly from fire. Based on this background information, we present an experimental design and the results of a fire experiment on bark samples that were used as a basis for assessing the fire tolerance of various tree species, enabling them to survive a surface fire characterized by temperatures up to 200 °C and 5 min duration. In Section 5, we give information on suitable bark samples to be investigated with the flaming test. In the Supplementary Materials File S1, we provide worksheets for lecturers, teachers, and students at high schools, colleges, and universities to analyze the fire tolerance of various tree bark samples.

## 2. Fire Landscapes of the Earth

The African savannas, tropical rain forests, the Mediterranean region, Australia, and California are amongst the regions in which wildfires occur regularly. In African savannas, human-caused fires are common at intervals of one to three years. Winter slash-and-burn fires turn grass and brush into nutrient-rich ash, which fertilizes the soil and promotes the growth of fresh grass for livestock in the spring. Over time, fire-resistant species become established because their seeds and roots are not damaged by fire. Slash-and-burn agriculture in tropical rain forests, combined with extreme droughts, has frequently led to uncontrolled large-scale forest fires in recent years, destroying millions of hectares of rain forest. In Europe, only the Mediterranean region belongs to the so-called fire landscapes (Figure 1a), with Mediterranean ecosystems that are highly fire-resilient (e.g., shrub-lands and oak forest) and some that are fire-sensitive (e.g., pine woodland) [20]. Australia is a continent that is repeatedly hit by catastrophic bush and forest fires. During 2003–2016, the largest fires were found in this continent [21]. After long periods of drought, fires caused by lightning and fanned by wind threaten flora, fauna, and people over large areas. Within the Australian tree population, 70% are eucalyptus species whose bark contains highly flammable oils. Eucalyptus bark is so light that burning material can be carried by the wind beyond the fire front, igniting spot fires many kilometers ahead of a bushfire [22]. Because of these “flying fires”, fires in Australia spread rapidly and are extremely difficult to control.

**Figure 3:**
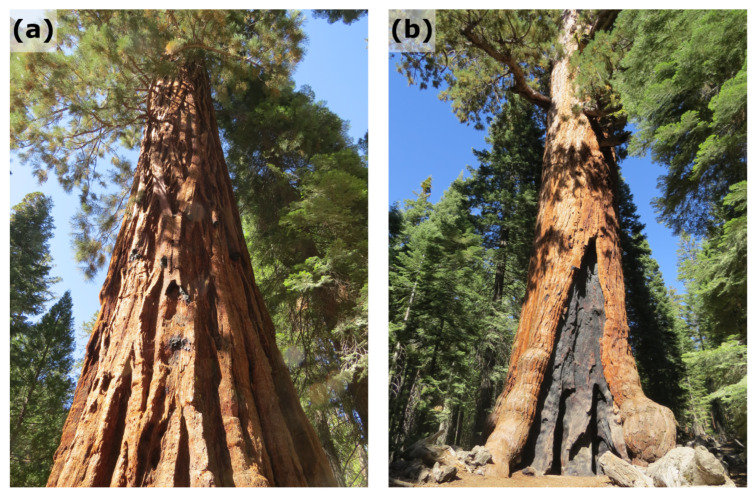
Giant sequoias (*Sequoiadendron giganteum*) in the Yosemite National Park, CA, USA. (**a**) The tree bark is undamaged, (**b**) the tree shows signs of fire damage but is still alive.

The forests of the Sierra Nevada in USA have experienced fires at intervals as rapid as 10 to 30 years. These surface fires have played an important role in the ecosystem, allowing trees, such as the impressive giant sequoias (*Sequoiadendron giganteum*, Figure 3), to survive and burning the undergrowth. In the early years of the Sequoia National Park, wildfires were immediately suppressed. In subsequent years, however, the population of young giant sequoias was found to be in decline. In 1968, Sequoia-Kings Canyon National Parks initiated an annual prescribed burning program together with a "let burn" policy that allowed lightning-caused fires to continue to burn if they posed no threat to resources. Since then, the area burned by lightning fires has been more than twice the area burned by human-caused fires [23].

## 3. Pyrophytes—Fire-Adapted Specialists in the Plant Kingdom

In biological systems, we distinguish between response, acclimation, and adaptation to environmental changes [5]. “Response” refers to the reaction of individual plants to an environmental stimulus within seconds to minutes. Immediate stress response is a reduction in physiological activity [5]. A prime example is the opening mechanism of the *Banksia* fruit in response to heat, which is frequently caused by fire [24]. “Acclimation”, however, is based on the non-hereditary alteration of growth patterns of individual plants by changed gene expression with respect to environmental changes over a period of days or weeks [5]. Periodic exposure to high but sub-lethal temperatures alters a plant’s physiology and morphology and increases its heat tolerance to temperatures that would kill it in the event of heat shock. Potential acclimation mechanisms to protect the photosynthetic apparatus and cellular metabolism include the synthesis of heat shock proteins and isoprene and antioxidant production [25]. “Adaptation”, in contrast, is based on a change of genetic information in populations of plants over evolutionary time [5]. In the case of fire-adapted trees, the variation of hereditary traits includes a thick and highly structured bark [26,27]. The thick bark insulates and protects the cambium, which is a cylinder of unspecified meristematic cells that lie inside the trunk and that form new tissues such as secondary xylem inwards and secondary phloem and bark outwards. However, if the temperature inside the trunk rises above 60 °C, the cambium dies, and so does the tree, because it cannot form new tissues [28,29]. Cork oaks (*Quercus suber*), many pine species (*Pinus* sp.), giant sequoias (*Sequoiadendron giganteum*) [26], and eucalyptus trees (*Eucalyptus obliqua*) [29] have an extremely high fire tolerance because of the thermal insulating properties of their bark. These wildfire-adapted plants are called pyrophytes because heat directly or indirectly flavors them. Direct benefits of wildfires include the heat-dependent opening of cones or fruits allowing seeds to be released. A prime example is the woody fruit of the Australian plant genus *Banksia* whose two valves split at an opening temperature ranging between 68 and 75 °C [24]. Indirect benefits of wildfires include released seeds falling onto the ash-fertilized soils, where they readily germinate and grow into young trees once the fire has destroyed more fire-sensitive competing plants.

## 4. Technical Applications

Since ancient times, bark preparations from various tree species have been used in folk medicine to cure diseases. Moreover, the burning of bark has been considered as alternative source of energy to wood. Bark is also employed as a mulch in agriculture and horticulture and to produce particle boards [30]. Nowadays, after simple and cheap treatments, bark can even be used for the fabrication of woven textiles [31]. The bark of the cork oak has, for centuries, provided thermal insulation on buildings. Today, cork and cork-based materials are used in the building industry with their favorable ecological footprint meeting green building demands [32]. The fields of application of bark range from the production of flame-retardant door panels [33] and thermal insulation for intercontinental ballistic missiles [34] to the sealing of wine bottles. However, tree bark can also provide ideas for biologically inspired engineered materials. As early as 1965, Hare suggested that bark, with its many air cells and its high content of cork, rich in phenolic compounds, could be considered as a “natural design for an insulating board” [35]. Some tree barks, such as those of giant sequoias and cork oaks, even have self-fire-extinguishing properties because of their high levels of phenolic compounds [36]. Thus, findings from further research into the influence of bark properties on its thermal insulation properties, combined with the known active principles of current thermal insulation materials, might stimulate the development of new biomimetic materials with improved thermal insulation properties.

## 5. Educational Module “Fire Tolerance of Various Tree Species”

The topic of “wildfires” and “forest fires” with its ambivalent character can be taught at high schools, colleges and universities by using the background information of this article and the working materials provided in the following text and in the Supplementary Materials File S1. In the previous sections, we have provided background information on wildfires that can be used by scientists, lecturers, teachers, and students as an introduction to this hot topic. The flaming experiments of bark samples described below can be used to investigate the fire tolerance of various tree species by scientists as part of a scientific project and by students within a practical laboratory course [26,27]. In addition, ecological aspects of the occurrence and significance of forest fires can be determined, and our basic knowledge of fire management can thereby be strengthened. The teaching concept presented is particularly suitable for (1) biology lessons on ecology (fire landscapes on Earth), evolution (fire-adapted traits), and the body plans of plants (especially trees), (2) for geography lessons on landscape units, biogeographical units, and economic geography in various climatic zones, and (3) for classes that combine the natural sciences and technology.

### 5.1. Independent Preparation by Students

Students can compile background information on wildfires and fire landscapes in the form of presentations or posters through independent research work in scientific publications and on the Internet. The Supplementary Materials File S1 “Information” provides students with essential information about the educational module and gives them support for the tasks presented in the Supplementary Materials File S1 “Research” for independent preparation. Students can, for example, list the various types of fire in the form of a table (cf. Table 1).

They can report on the advantages and disadvantages of wildfires (cf. Section 1) or present the origin, consequences, and prevention of forest fires regions that have higher or lower fire risk (cf. Section 2).

To gain a better understanding of the relationship between the fire tolerance of trees and their occurrence in low, medium, or high fire-prone areas, the students should profile the six proposed tree species, e.g., in the form of a table (cf. Table 2). We recommend choosing these tree species for the study, as we can present results for them as well. The distribution of the species, also in the form of a distribution map, can be easily looked up in plant identification books or on the Internet and then compared with maps of fire regimes. Lavorel et al. [37] present a global map of estimated fire regimes that displays predominant cause (e.g., natural, human), type (e.g., surface fire, crown fire), and frequency of fire for FAO (=Food and Agriculture Organization) ecological zones. The fire frequency relates to the fire cycle of more than 200 years (low frequency), 20 to 200 years (medium frequency) and less than 20 years (high frequency). San-Miguel and Camia [38] provide a European map of the average annual distribution of the numbers of fires. Both figures, namely Figure 2 in [37] and Figure 3 in [38], can be used to compare the fire frequency with the distribution areas of the tree species studied and thus facilitate an assessment of low, medium and high fire frequency. See also Supplementary Materials File S1 “Research”.

**Figure 4:**
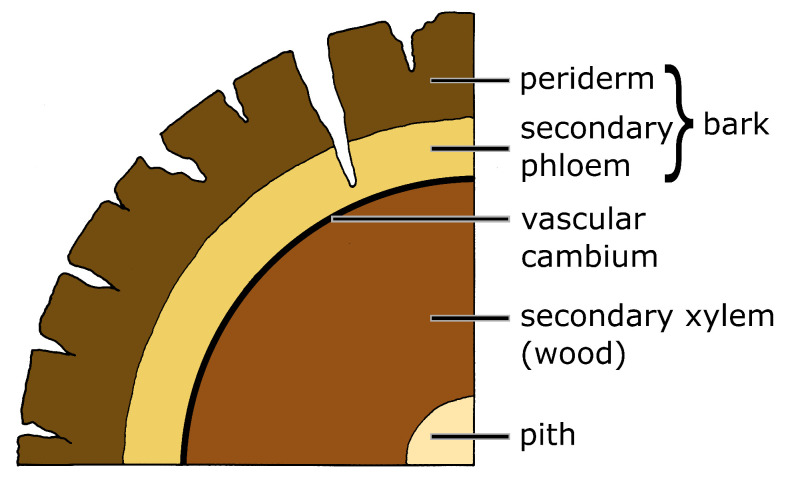
Schematic drawing of the cross-section of a tree trunk (not to scale). The vascular cambium is the layer of tissue in which cell division takes place. It forms secondary xylem (wood) inwards. All tissue layers outside the vascular cambium are referred to as bark and include the secondary phloem and the periderm. The periderm is a secondary covering that consists (from the outside to the inside) of cork (phellem), cork cambium (phellogen) and living cells (phelloderm). The bark has a surface structuring with indentations of various depths (Adapted from [27]).

Students should also become familiar with the thickness and structure of the bark, which is essential for fire tolerance. The structure of bark is complex and consists of several layers of tissue. This might be confusing at first glance, but in this educational module, we will consider the bark as the totality of all the tissues outside of the cambium and thus as a functional unit. In the event of a fire, the bark functions as the outermost protection layer from the impact of the flames. Bark is a nontechnical term and refers to all the tissues outside the vascular cambium. Young trees have a thin primary bark, which consists from the outside to the inside of an epidermis (the latter may be replaced by periderm), cortical cells, primary and secondary phloem, which is followed toward the inside by a vascular cambium, secondary and primary xylem (wood), and a pith. In old trees, however, various dermal tissues develop at different depths during secondary cortical growth (Figure 4). These tissues are formed by meristematic cells capable of division in the so-called cambia, which is located in various areas of the trunk periphery. The vascular cambium is the main tissue causing secondary growth in the trunk and produces wood (secondary xylem) inwards, toward the pith, and secondary phloem outwards, toward the periderm. In the area of the periderm, the re-embryonalization of living cells can form a cork cambium (phellogen), which can produce a thick layer of cork cells outwards (phellem) and living cells (phelloderm) inwards [36]. See also the Supplementary Materials File S1 “Research”.

### 5.2. Materials and Methods

#### 5.2.1. Bark Samples

The lecturer or teacher should obtain suitable bark samples either alone or with the students. In our experiences, foresters or staff from sawmills or local authorities are extremely helpful regarding the need for bark samples for educational purposes. Samples can be collected within a few weeks of a suitable tree being available or felled in the area. Bark samples of cork oak are also available from specialized aquarium dealers.

Bark samples from giant sequoia (*Sequoiadendron giganteum* (Lindl.) J. Buchholz), cork oak (*Quercus suber* L.), Scots pine (*Pinus sylvestris* L.), European larch (*Larix decidua* Mill.), European beech (*Fagus sylvatica* L.), and silver fir (*Abies alba* Mill.) are suitable for the flaming experiment (Table 2), for which we can present individual results. The samples should be about 10 cm × 20 cm in size and extend to the cambium on the inside of the secondary xylem, i.e., the sample should include the entire bark (cf. Figure 4). The latter is easy to obtain as, when peeled off, the bark usually separates from the wood of the trunk at the cambium. All samples should be stored in a dry room for about a week so that they have approximately the same moisture content [27].

#### 5.2.2. Flaming Tests

If the meristematic cells of the vascular cambium are exposed to lethal temperatures of 60 °C and higher, the tree will die because it will not be able to form new cells, and thus, secondary growth and bud burst are stopped, both of which are essential for living trees [28,29]. Therefore, the time ($\tau_{60}$) needed for the vascular cambium to reach 60 °C is a measure of the fire tolerance of a tree and of its ability to survive a surface fire at approximately 200 °C and of 5 min duration. The time $\tau_{60}$ can be determined in the laboratory by using a simple flaming experiment on bark samples [26]. The experimental investigation of the fire tolerance of bark samples can be carried out by teams of at least three students from 15 years onwards.

**Figure 5:**
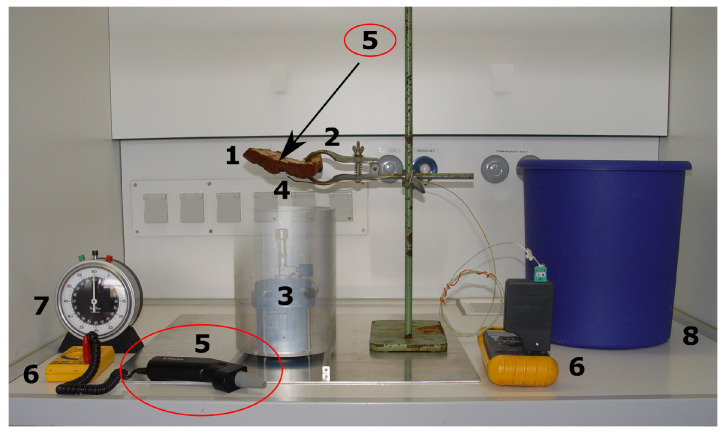
Experimental set-up for fire-tolerance analyses in the fume cupboard. A bark sample (1) is clamped (2) at a height of 43 cm over (3) a Bunsen burner with a flame length of about 20 cm. Additionally, the Bunsen burner can be protected by a hollow aluminum cylinder (indicated as “see-through” in the picture). The flame temperature is measured by (4) a thermocouple, and the temperature of the inner bark surface, i.e., the position of the cambium in the living tree, is measured in a contactless manner by (5) an infrared thermometer (position indicated). Data are recorded via (6) a computer. Time is measured by (7) a stopwatch. As a precaution, (8) a bucket of water should be available to throw in any bark samples that catch fire.

The flaming experiment (Figure 5) is carried out under a fume cabinet. Alternatively, a hollow aluminum cylinder can be placed over a Bunsen burner to avoid any disturbing draughts during the test. A bucket of water should be provided as a precaution, although barks that catch fire during the experiment usually do not continue to burn when removed from the flame. The bark sample is clamped over a Bunsen burner with the outer surface facing toward the flame. A thermometer with an external sensor measures the temperature of the flame just below the bark. An infrared thermometer measures the temperature on the inner sample surface where the thin, sensitive cambium is located. Temperatures of the flame and the inner bark surface are recorded by means of a computer. The experiment either ends as soon as the inner bark surface exceeded 60 °C or after 5 min if 60 °C is not reached [26,27].

### 5.3. Results

**Figure 6:**
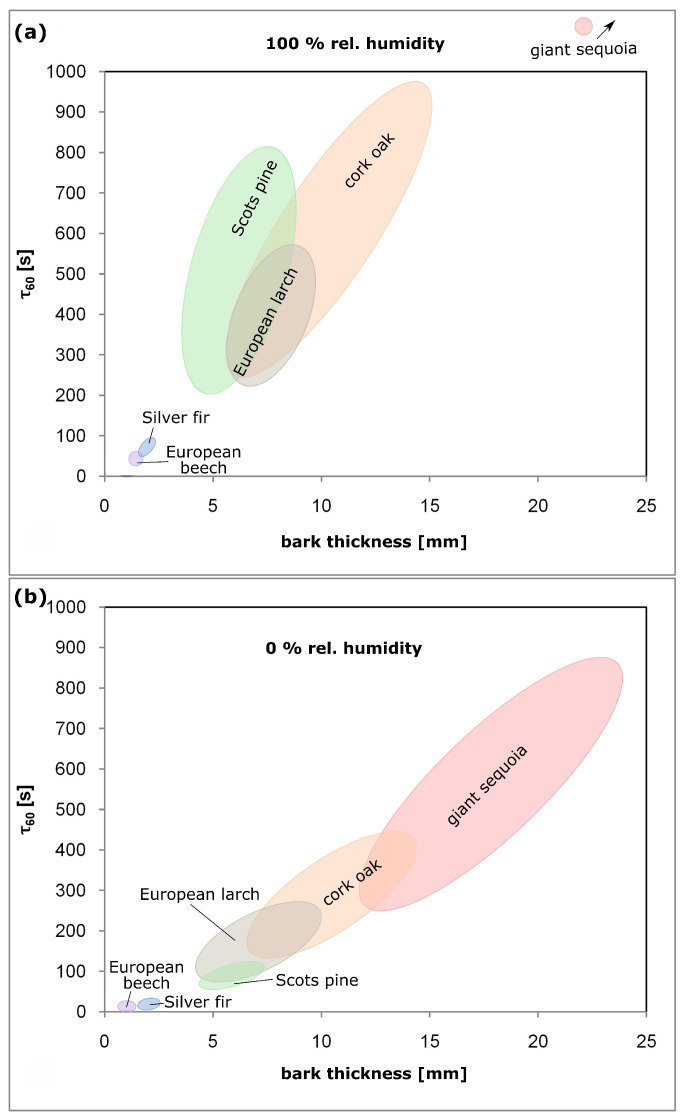
Fire tolerance of various bark samples. The time $\tau_{60}$ is given as a function of bark thickness. (**a**) Samples were stored at 100% relative humidity. The values for the giant sequoia are not shown within the graph because all samples had not reached the temperature of 60 °C after 5 min. This is indicated by the arrow. (**b**) Samples that were dried and stored at 0% relative humidity.

Studies by Bauer et al. [26] have shown that for a given bark moisture content, the fire tolerance of a tree can be inferred from the bark thickness alone (Figure 6). For all tree species that we studied, the $\tau_{60}$ of the wet samples (stored at 100% relative humidity) was significantly higher than the $\tau_{60}$ of the dry samples (stored at 0% relative humidity) [26]. This was also the case for samples of the giant sequoia, whose $\tau_{60}$ values in wet samples were so high that some tests were terminated after 21 min. Flame temperatures averaged 214.6 °C ± 20.2 °C, which is within the temperature range characteristic of surface fires (cf. Table 1).

**Figure 7:**
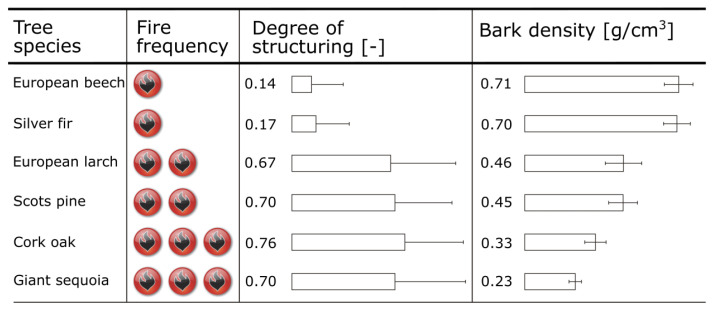
Frequency of forest fires in the natural habitats of the studied tree species are given together with the mean values of the degree of structuring and of the density of their bark. The bar chart displays the mean values and the standard deviations.

Other bark properties such as density and degree of structuring also show a correlation with fire tolerance, but their importance in determining the extent of fire tolerance appears to be secondary to the influence of bark thickness and bark moisture (Figure 7). Bark density in terms of mass per volume was measured by xylometric methods [45]. Surface structuring of the bark is depicted in Figure 4. The degree of structuring is calculated from the maximum and minimum bark thicknesses and the thicknesses at two randomly chosen points of the bark sample [26]. The higher the value, the more pronounced the surface structuring.

## 6. Conclusions

Wildfires can be both a blessing for fire-adapted plants (phyrophytes) in Earth’s fire landscapes and a curse for human society in civilized areas. With this article, we would like to make a contribution to this ambivalent topic for science as well as for education and teaching. In the context of climate change, with rising temperatures, decreasing soil moisture, and drier organic matter as potential fuels, we need a better understanding of the increasing risk and extent of wildfires and the potential direct and indirect benefits of fire-adapted trees for forest survival in the event of fire. Forest fires have been monitored worldwide for decades and show annual variations in the number of fires and the area burned. The various forest fires differ in temperature and duration and thus in the damage to vegetation. Even fire-adapted trees with a thick bark can only survive surface fires having a temperature of 200 °C and a passage duration of 5 min. Cork oaks, many pine species, eucalyptus trees, and, in particular, giant sequoias have an extremely high fire tolerance because of the thermal insulating properties of their bark, which protects the meristematic cells of the vascular cambium from lethal temperatures above 60 °C. These results are consistent with those of [29], who identified 60 °C as the critical temperature for cambium cells of *Eucalyptus obliqua*.

The topic of “wildfires” and “forest fires” with their ambivalent character can be taught at high schools, colleges or universities. We provide this article as extended background information to the four working sheets presented in the Supplementary Materials File S1. Our focus is on the flaming test of various bark samples to quantify their fire tolerance. Fire-tolerant trees possess bark capable of insulating the vascular cambium from critical temperatures above 60 °C for a period of 5 min. From our results, we assume that the fire tolerance of a tree species depends on its ability to build up a thick bark layer as quickly as possible with the degree of structuring and the density of the bark playing minor roles.

## Figures and Tables

**Table 1:** Description of various fire types and their effect on vegetation.

Fire Type	Description	Temperature	Combusted Material	Effect on Vegetation
Ground fires	flameless, smoldering, burn slowly (days to months)	“hot fire”, >500 °C	organic materials in the soil and on the surface	externally intact trees die because of burned root systems
Surface fires	burn rapidly (seconds to minutes)	“cold fire”, up to 200 °C	surface and undergrowth	trees survive and regrow, bark is destroyed, vascular cambium remains functional
Crown fires	only tree crowns burn	“hot fire”, >1000 °C	material at canopy level burns	trees die
Full fires	burn slowly (days to months)	“hot fire”, up to 500 °C	combination of surface and crown fire	complete stand destruction
Flying fires	burning plant structures fly over the fire front and cause new fires	dependent on the type of fire that is triggered	dependent on the causing fire	can ignite new fires

**Table 2:** Overview on the selected tree species and their natural distribution in areas of high, medium, or low fire risk [26,27].

Tree Species	Latin Name	Natural Distribution Area	Forest Fire Frequency
Giant sequoia	*Sequoiadendron giganteum* (Lindl.) J. Buchholz	California [39]	high
Cork oak	*Quercus suber* L.	Mediterranean region [40]	high
Scots pine	*Pinus sylvestris* L.	Europe, North Asia [41]	medium
European larch	*Larix decidua* Mill.	Central Europe [42]	medium
European beech	*Fagus sylvatica* L.	West and Central Europe [43]	low
Silver fir	*Abies alba* Mill.	West, South, and Central Europe [44]	low

## Data Availability

All relevant data are included within the paper and its Supplementary Materials Files S1 and S2.

## Supplementary Materials

The following supporting information can be downloaded at https://www.mdpi.com/article/10.3390/biomimetics9020114/s1, File S1: Educational module “Fire Tolerance of Various Tree Species”; File S2: Data of wildfires in North America and Europe.

## References

[1] European Commission The EU 2022 Wildfire Season Was the Second Worst on Record. https://joint-research-centre.ec.europa.eu/jrc-news-and-updates/eu-2022-wildfire-season-was-second-worst-record-2023-05-02_en.

[2] Fernandez-Anez N., Krasovskiy A., Müller M., Vacik H., Baetens J., Hukić E., Kapovic Solomun M., Atanassova I., Glushkova M., Bogunović I. (2021). Current wildland fire patterns and challenges in Europe: A synthesis of national perspectives. Air Soil Water Res..

[3] Pausas J.G., Keeley J.E. (2019). Wildfires as an ecosystem service. Front. Ecol. Environ..

[4] Pausas J.G., Schwilk D. (2012). Fire and plant evolution. New Phytol..

[5] Lambers H., Chapin F.S., Pons T.L. (2008). Plant Physiological Ecology.

[6] Schultz M.G., Heil A., Hoelzemann J.J., Spessa A., Thonicke K., Goldammer J.G., Held A.C., Pereira J.M., van Het Bolscher M. (2008). Global wildland fire emissions from 1960 to 2000. Glob. Biogeochem. Cycles.

[7] World Health Organization Wildfires. https://www.who.int/health-topics/wildfires#tab=tab_1.

[8] San-Miguel-Ayanz J., Durrant T., Boca R., Libertà G., Branco A., de Rigo D., Ferrari D., Maianti P., Artès V.T., Duarte O. (2022). Advance Report on Wildfires in Europe, Middle East and North Africa 2021 EUR 31269 EN.

[9] National Interagency Coordination Center (2022). Wildland Fire Summary and Statistics Annual Report 2022. https://www.nifc.gov/sites/default/files/NICC/2-Predictive%20Services/Intelligence/Annual%20Reports/2022/annual_report.2.pdf.

[10] National Interagency Fire Center Total Wildland Fires and Acres (1983–2022). https://www.nifc.gov/fire-information/statistics/wildfires.

[11] European Environment Agency Burnt Area in European Countries. https://www.eea.europa.eu/data-and-maps/daviz/burnt-forest-area-in-five-4#tab-chart_5.

[12] Moreira F., Viedma O., Arianoutsou M., Curt T., Koutsias N., Rigolot E., Barbati A., Corona P., Vaz P., Xanthopoulos G. (2011). Landscape—Wildfire interactions in southern Europe: Implications for landscape management. J. Environ. Manag..

[13] WWF Germany Forets Ablaze—Causes and Effects of Global Forest Fire. https://www.wwf.de/fileadmin/fm-wwf/Publikationen-PDF/WWF-Study-Forests-Ablaze.pdf.

[14] National Interagency Fire Center Human-Caused Wildfires (2001–2022). https://www.nifc.gov/fire-information/statistics/human-caused.

[15] National Interagency Fire Center Lightning-Caused Wildfires (2001–2022). https://www.nifc.gov/fire-information/statistics/lightning-caused.

[16] Vilar L., Camia A., San-Miguel-Ayanz J., Martín M.P. (2016). Modeling temporal changes in human-caused wildfires in Mediterranean Europe based on Land Use-Land Cover interfaces. For. Ecol. Manag..

[17] Mhawej M., Faour G., Adjizian-Gerard J. (2015). Wildfire likelihood’s elements: A literature review. Challenges.

[18] Richter M. (1997). Allgemeine Pflanzengeographie.

[19] WClimateCheck Wildfire Types and Their Risk to You and Your Home. https://climatecheck.com/risks/fire/types-of-wildfire.

[20] Pausas J.G., Llovet J., Rodrigo A., Vallejo R. (2008). Are wildfires a disaster in the Mediterranean basin?—A review. Int. J. Wildland Fire.

[21] Andela N., Morton D.C., Giglio L., Paugam R., Chen Y., Hantson S., Van Der Werf G.R., Randerson J.T. (2019). The Global Fire Atlas of individual fire size, duration, speed and direction. Earth Syst. Sci. Data.

[22] Hall J., Ellis P.F., Cary G.J., Bishop G., Sullivan A.L. (2015). Long-distance spotting potential of bark strips of a ribbon gum (*Eucalyptus viminalis*). Int. J. Wildland Fire.

[23] Keeley J.E., Pfaff A., Caprio A.C. (2021). Contrasting prescription burning and wildfires in California Sierra Nevada national parks and adjacent national forests. Int. J. Wildland Fire.

[24] Huss J.C., Spaeker O., Gierlinger N., Merritt D.J., Miller B.P., Neinhuis C., Fratzl P., Eder M. (2018). Temperature-induced self-sealing capability of Banksia follicles. J. R. Soc. Interface.

[25] Kozlowski T.T., Pallardy S.G. (2002). Acclimation and adaptive responses of woody plants to environmental stresses. Bot. Rev..

[26] Bauer G., Speck T., Blömer J., Bertling J., Speck O. (2010). Insulation capability of the bark of trees with different fire adaptation. J. Mater. Sci..

[27] Speck O., Bauer G., Speck T. (2012). Naturkatastrophe Waldbrand? Untersuchung der Feuertoleranz bei verschiedenen Baumarten. Prax. Nat. Biol..

[28] Wade D.D. (1993). Thinning young loblolly pine stands with fire. Int. J. Wildland Fire.

[29] Subasinghe Achchige Y.M., Volkova L., Drinnan A., Weston C.J. (2021). A quantitative test for heat-induced cell necrosis in vascular cambium and secondary phloem of *Eucalyptus obliqua* stems. J. Plant Ecol..

[30] Pasztory Z., Mohacsine I.R., Gorbacheva G., Börcsök Z. (2016). The utilization of tree bark. BioResources.

[31] Wenig C., Dunlop J.W., Hehemeyer-Cürten J., Reppe F.J., Horbelt N., Krauthausen K., Fratzl P., Eder M. (2021). Advanced materials design based on waste wood and bark. Philos. Trans. R. Soc..

[32] Pereira H., Knapic S. (2017). Bark and cork. Performance of Bio-Based Building Materials.

[33] Megraw R.A. (1976). Preparation of a Three Layer, Fire Retardant Particleboard. U.S. Patent.

[34] Hovey R.W. (1965). Cork thermal protection design data for aerospace vehicle ascent flight. J. Spacecr. Rocket..

[35] Hare R.C. (1965). Contribution of bark to fire resistance of southern trees. J. For..

[36] Leite C., Pereira H. (2017). Cork-containing barks—A review. Front. Mater..

[37] Lavorel S., Flannigan M.D., Lambin E.F., Scholes M.C. (2007). Vulnerability of land systems to fire: Interactions among humans, climate, the atmosphere, and ecosystems. Mitig. Adapt. Strateg. Glob. Chang..

[38] San-Miguel J., Camia A. (2009). Forest fires at a glance: Facts, figures and trends in the EU. Living with Wildfires: What Science Can Tell Us—A Contribution to the Science-Policy Dialogue.

[39] Sillett S.C., Van Pelt R., Carroll A.L., Campbell-Spickler J., Antoine M.E. (2019). Structure and dynamics of forests dominated by *Sequoiadendron giganteum*. For. Ecol. Manag..

[40] Durrant T.H., De Rigo D., Caudullo G. (2016). *Quercus suber* in Europe: Distribution, habitat, usage and threats. Eur. Atlas For. Tree Species.

[41] Aas G. (2007). Systematik, Verbreitung und Morphologie der Waldkiefer (*Pinus sylvestris*). LWF Wissen.

[42] Aas G. (2012). Die Europäische Lärche–Taxonomie, Verbreitung, Morphologie. LWL Wissen.

[43] Aas G. (2022). Die Rotbuche (*Fagus sylvatica*): Verwandtschaft, Morphologie, Verbreitung und Ökologie. LWF Wissen.

[44] Aas G. (2004). Die Weißtanne (*Abies alba*)—Dendrologische Anmerkungen. LWF Wissen.

[45] Özçelik R., Wiant H.V., Brooks J.R. (2008). Accuracy using xylometry of log volume estimates for two tree species in Turkey. Scand. J. For. Res..

