# Supplementary material for: Is a Forest Fire a Natural Disaster? Investigating the Fire Tolerance of Various Tree Species—An Educational Module
Source: Biomimetics (Basel). 2024 Feb 15;9(2):114. doi: 10.3390/biomimetics9020114 (PMC10887004; doi:10.3390/biomimetics9020114)
Supplement: Supplementary file 1 [file biomimetics-09-00114-s001.zip › S1_fire-tolerance.pdf]

biomimetics

Supplementary Material S1

Is a Forest Fire a Natural Disaster? Investigating the Fire Tolerance of Various Tree Species—An Educational Module

Olga Speck [1,2,*] and Thomas Speck [1,2]

[1] Plant Biomechanics Group @ Botanic Garden Freiburg, University of Freiburg, D-79104 Freiburg, Germany; olga.speck@biologie.uni-freiburg.de (O.S.); thomas.speck@biologie.uni-freiburg.de (T.S.)
[2] Cluster of Excellence *liv*MatS @ FIT – Freiburg Center for Interactive Materials and Bioinspired Technologies, D-79110 Freiburg, Germany; olga.speck@biologie.uni-freiburg.de (O.S.); thomas.speck@biologie.uni-freiburg.de (T.S.)
* Correspondence: olga.speck@biologie.uni-freiburg.de; Tel.: +49-761-203-2803

Educational Module: "Fire Tolerance of Various Tree Species"

The presented module is based on scientific research in the Plant Biomechanics Group of the University Freiburg, Germany. This module is suitable for students from 15 years onwards. For safety reasons, an adult should be present when the flaming tests are carried out. The degree of difficulty is "medium". The experiment for 6 samples takes 6 x 25 minutes.

The following instructions are addressed directly to the students of high schools, colleges, or universities. The instructions are divided into six parts:

1. Information: Fire tolerance of trees trunks
2. Research: Tasks for independent preparation
3. Experiment: Flaming test of bark samples
4. Solution: Answers to all tasks

Publisher's Note: MDPI stays neutral with regard to jurisdictional claims in published maps and institutional affiliations.

Copyright: © 2021 by the authors. Submitted for possible open access publication under the terms and conditions of the Creative Commons Attribution (CC BY) license (https://creativecommons.org/licenses/by/4.0/).

Fire tolerance of tree trunks

Fire tolerance of tree trunks—Thanks to their thick layers of bark, giant sequoias and cork oaks are more fire tolerant than other trees. Although other properties of bark, such as water content, density, or surface structuring, also play a role, those trees that are capable of forming extremely thick layers of bark have had great advantages attributable to their adaptations to fire during biological evolution. A thick layer of bark is a fire-adaptive trait because the bark protects the underlying cambium, a layer of living cells, each of which can divide forming bark on the outside and wood on the inside. Thus, if the meristematic cambium cells of the cambium are exposed to a lethal temperature of 60°C by fire, the cells die and so does the tree.

Fire types—Wildfires are unplanned fires perceived as a threat by humans. However, fires are essential for the survival of fire-adapted plants. On the one hand, wildfires cause major damage worldwide, burning large areas of forests and landscapes, threatening towns and villages, and generating high levels of air pollution. On the other hand, fire-adapted plants (pyrophytes) occur in the fire landscapes of the Earth and can survive exposure to heat (e.g., because of having thick bark that protects living tissue). They also benefit from fire directly (e.g., fire initiates cone opening and seed release) or indirectly (e.g., fewer competing plants of other species remain, and seeds germinate in the ash-fertilized soil).

Fire-adapted barks—The different types of forest fires can have various effects on vegetation. Trees cannot survive so-called hot fires with temperatures > 500 °C. However, they can survive cold fires, such as surface fires, if their bark is thick enough to protect the meristematic cells of the vascular cambium. Thus, fire-adapted trees have bark that can insulate the cambium from temperatures > 60°C for a period of 5 minutes. Interestingly, the density in terms of mass per volume and the surface structuring in terms of indentations of various depth of the bark correlate with fire tolerance, but do not play a significant role for fire tolerance (Bauer et al. 2010).

Dermal tissues of tree trunks—Bark is a non-technical term that groups all tissue layers outside the vascular cambium with meristematic cell that form cells outwards and inwards (Figure 1).

Fire tolerance of tree trunks

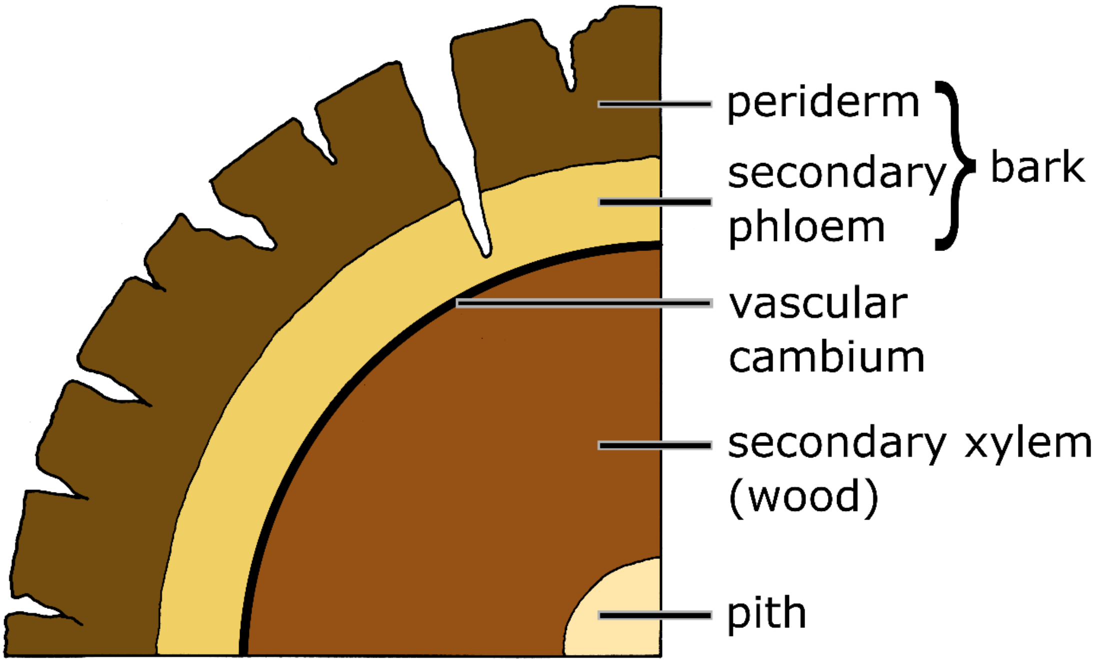

Figure S1: Schematic drawing of the cross-section of a tree trunk (not to scale). The vascular cambium is the layer of tissue in which cell division takes place. It forms secondary xylem (wood) inwards. All tissue layers outside the vascular cambium refer to the bark, which includes the secondary phloem and the periderm. The periderm is a secondary covering that consists, from the outside to the inside, of cork, cork cambium, and phelloderm. The surface structuring is depicted by indentations of various depth. From Speck et al. 2012.

References:

Bauer, G.; Speck, T.; Blömer, J.; Bertling, J.; Speck, O. Insulation capability of the bark of trees with different fire adaptation. *Journal of Materials Science* **2010**, *45*, 5950–5959.

Speck, O.; Bauer, G.; Speck, T. Naturkatastrophe Waldbrand? Untersuchung der Feuertoleranz bei verschiedenen Baumarten. *Prax. Nat. Biol.* **2012**, 61, 36–44 (in German).

Fire tolerance of tree trunks

Task 1: Fire types and fire landscapes

a) Make a table of the different types of fire with a description of the fire, temperature, combusted material, and effect on vegetation.

Fire type	Description of the fire	Temperature	Combusted material	Effect on vegetation

b) Name five fire landscapes.

____

____

____

____

____

c) List various causes of forest fires.

____

____

____

____

____

Task 2: Fire tolerance

a) Define the term "fire tolerance".

____

____

____

____

____

b) How can “fire tolerance” be measured quantitatively?

____

____

____

____

____

c) Name two fire-tolerant tree species and their natural habitat.

____

____

d) Describe the direct and indirect benefits of wildfire for vegetation by using the giant sequoia as an example.

Task 3: Dermal tissue of tree trunks

a) Label the trunk cross-section (image from Speck et al. 2012).

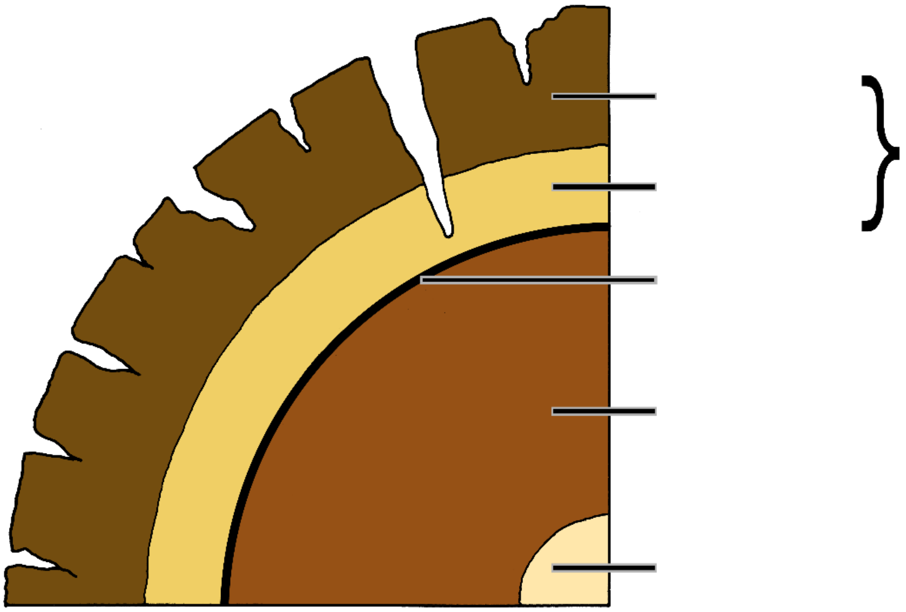

b) Name the tissue that needs special protection in the event of a forest fire.

Reference: Speck, O.; Bauer, G.; Speck, T. Naturkatastrophe Waldbrand? Untersuchung der Feuertoleranz bei verschiedenen Baumarten. *Prax. Nat. Biol.* **2012**, 61, 36–44 (in German).

Fire tolerance of tree trunks

We can measure the fire tolerance of tree species by a flaming test that records the time it takes until a temperature of 60°C is reached at the vascular cambium.

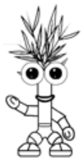	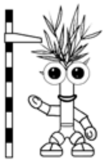	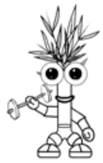	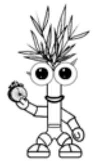
**Working type:** partner or group work with 3 students	**Age:** students older than 15 years & 1 adult	**Degree of difficulty:** medium	**Duration:** 1 week (storage of samples) & 6 x 25 minutes

Test samples— Bark samples from giant sequoia, cork oak, Scots pine, European larch, European beech, and silver fir are suitable for the flaming experiment. The samples should be about 10 cm x 20 cm in size and extend to the cambium on the inside of the secondary xylem, i.e., the sample should include the entire thickness of the bark. As the bark usually separates from the wood of the trunk at the cambium when it is peeled off, this is easy to achieve. All samples should be stored in a dry room for about a week so that they have approximately the same moisture content. Samples can be obtained from a forester or sawmill, and cork oak can be bought from aquarium shops.

Task 4: Create a table of the selected tree species, their natural distribution, and the frequency of forest fires (high, medium, low).

Experimental set up of the flaming test—The experiment is carried out within a fume cabinet (Figure S2). A hollow aluminum cylinder can be placed over the Bunsen burner to avoid any disturbing draughts during the experiment. The bark sample is clamped so that the Bunsen burner flame plays on the outside of the bark. A thermometer with an external sensor measures the temperature of the flame just below the bark. An in-frared thermometer is used to measure the temperature on the inside of the sample, where the thin sensitive cambium is located. A bucket of water should be provided as a precaution, although bark that burns during the experiment will usually not continue to burn when removed from the flame.

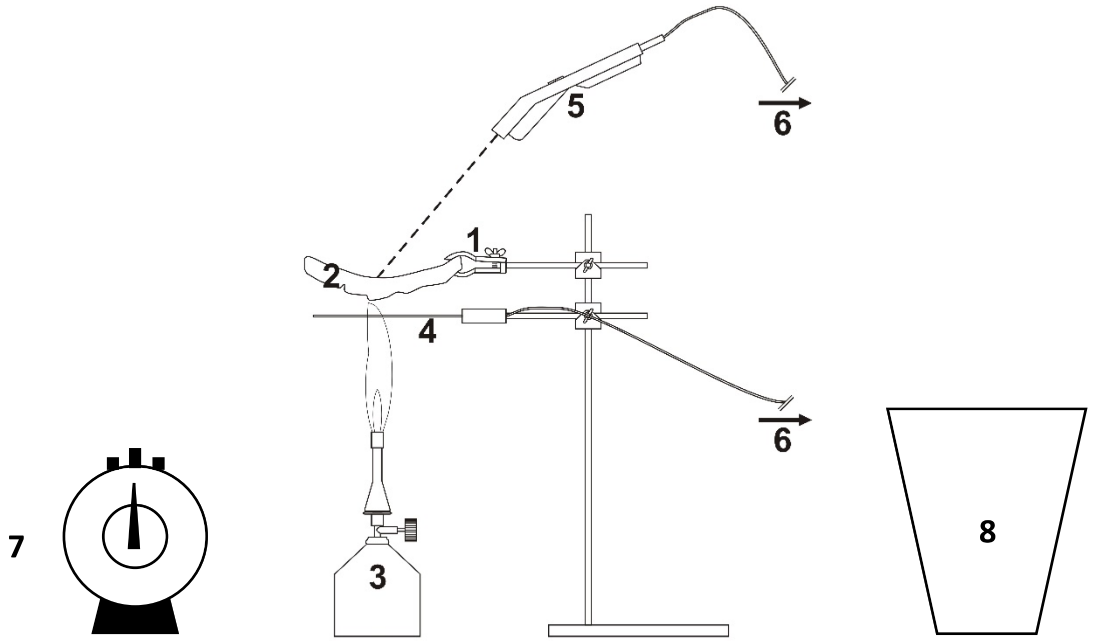

Figure S2: Schematic drawing of the experimental set up (1) Clamp, (2) Bark sample, (3) Bunsen burner, (4) Thermocouple to measure flame temperature, (5) Infrared thermometer to measure (without contact) the temperature of the inner bark surface, i.e., at the position of the cambium in the living tree, (6) Temperature data are recorded, (7) Stopwatch, (8) Bucket with water. (Adapted from Bauer et al. 2010).

Experimental procedure—The experimental investigation of fire tolerance of bark samples can be carried out by teams of at least three school pupils or university students. At least one adult should be a member of the team. The results are entered in the raw data table.

Initially, the thickness of each sample is measured at four different points by using a calliper. One student pushes the laboratory stand containing the bark sample into the flame, presses the stopwatch, and announces the time at the intervals specified in the raw data table. The second student measures the temperature values of both the bark and the flame and announces the values at the specified times. The third student records both the bark and flame temperatures in the raw data table. The test ends when either a temperature of 60°C (τ_{60}) is reached on the inside of the bark or 5 minutes have elapsed or the bark catches fire. The 'Remarks' box can be used to record, for example, when and if 60°C was reached, if there were traces of fire on the bark at the end of the test, or if the bark caught fire.

Table S1: Raw data

Sample #						
Tree species						
Thickness of sample (cm)						
Time (s)	T_{bark} [°C]	T_{flam} [°C]	T_{bark} [°C]	T_{flame} [°C]	T_{bark} [°C]	T_{flame} [°C]
0						
10						
20						
30						
40						
50						
60						
90						
120						
150						
180						
210						
240						
300						
Remarks						

Information

Research

Experiment

Solutions

Task 5: Explain why you can stop the experiment after 5 minutes.

Task 6: Explain why you are recording the flame temperature.

Results of the experiment— The raw data from Table S1 can be analyzed in the form of diagrams and statistical calculations after the experiment has been completed. This can be done using a spreadsheet editor such as Excel.

Task 7: Plot the temperature at the inner side of the bark T_{bark} [°C] on the y-axis as a function of the time [s] on the x-axis. You can also use a spreadsheet to do this. Discuss the results.

____

____

____

____

____

____

____

Task 8: Calculate the mean sample thickness for each sample. Plot the time [s] to critical temperature 60°C (τ_{60}) on the y-axis as a function of the mean sample thickness [cm] for each tree species. You can use a spreadsheet program to calculate the mean and to create the diagram. To determine the quantitative relationship between sample thickness and τ_{60}, you can include a trend line with a coefficient of determination that can have values between 0 and 1. If all values lie on the trend line, then a linear relationship exists, and R^2 = 1. If no linear relationship exists, then R^2 = 0. The closer the coefficient of determination is to 1, the more likely it is that a linear relationship exists. Discuss the results.

____

____

____

____

____

____

____

Fire tolerance of tree trunks

Task 1: Fire types and fire landscapes

a) Make a table of the different types of fire with a description of the fire, temperature, combusted material, and effect on vegetation.
See Table 1 in the main text.

b) Name five fire landscapes.
(1) African savannas, (2) tropical rain forests, (3) California, (4) Australia, (5) Mediterranean region

c) List various causes of forest fires.
Natural causes (4% of all forest fires): extreme weather events (high temperatures, drought, and storms), lightning, or volcanic eruptions.
Anthropogenic causes (96% of al forest fires): Campfires, cigarettes, matches, slash-and-burn agriculture, arson.

Task 2: Fire tolerance

a) Define the term "fire tolerance".
Fire tolerance is defined as the ability of a tree to survive a surface fire (up to 200°C and 5 minutes passage duration).

b) How can "fire tolerance" be measured quantitatively?
The measure of the fire tolerance for a tree species is the time taken within which the vascular cambium under the insulating bark reaches the critical temperature of 60°C.

c) Name two fire-tolerant tree species and their natural habitat.
(1) Giant sequoia (California), (2) Cork oak (Mediterranean region)

d) Describe the direct and indirect benefits of wildfire to vegetation by using the giant sequoia as an example.
Direct benefits: thick bark protects the living tissue from heat and enables survival of the tree from a fire, cone opening, and seed release

Indirect benefits: fewer competing plants of remaining fire-sensitive species, seeds germinate in the ash-fertilized soil, fire kills animal pests

Task 3: Dermal tissue of tree trunks

a) Label the trunk cross-section (image from Speck et al. 2012).
See Figure 4 in the main text or Figure S1 in the working sheet "Information".

b) Name the tissue that needs special protection in the event of a forest fire.
Vascular cambium

Task 4: Create a table of the selected tree species, their natural distribution, and the frequency of forest fires (high, medium, low).
See Table 2 in the main text.

Task 5: Explain why you can stop the experiment after 5 minutes.
The selected time of 5 minutes corresponds to the duration of most surface fires.

Task 6: Explain why you are recording the flame temperature.
The recording of the flame temperature is used to check that the experiment is carried out at a constant temperature.

Fire tolerance of tree trunks

Task 7: Plot the temperature at the inner side of the bark T_{bark} [°C] on the y-axis as a function of the time [s] on the x-axis. You can also use a spreadsheet to do this. Discuss the results.

**Silver fir (*Abies alba*)**		**Giant sequoia (*Sequoiadendron giganteum*)**	
Time [s]	T_{bark} [°C]	Time [s]	T_{bark} [°C]
0	23.5	0	22.2
10	29.5	10	23.1
20	32.4	20	23.9
30	38.2	30	25.1
40	42.0	40	25.9
50	49.8	50	26.0
60	55.5	60	27.6
67	60.7	90	28.3
		120	30.6
		150	33.8
		180	41.7
		210	47.5
		240	48.5
		300	55.0

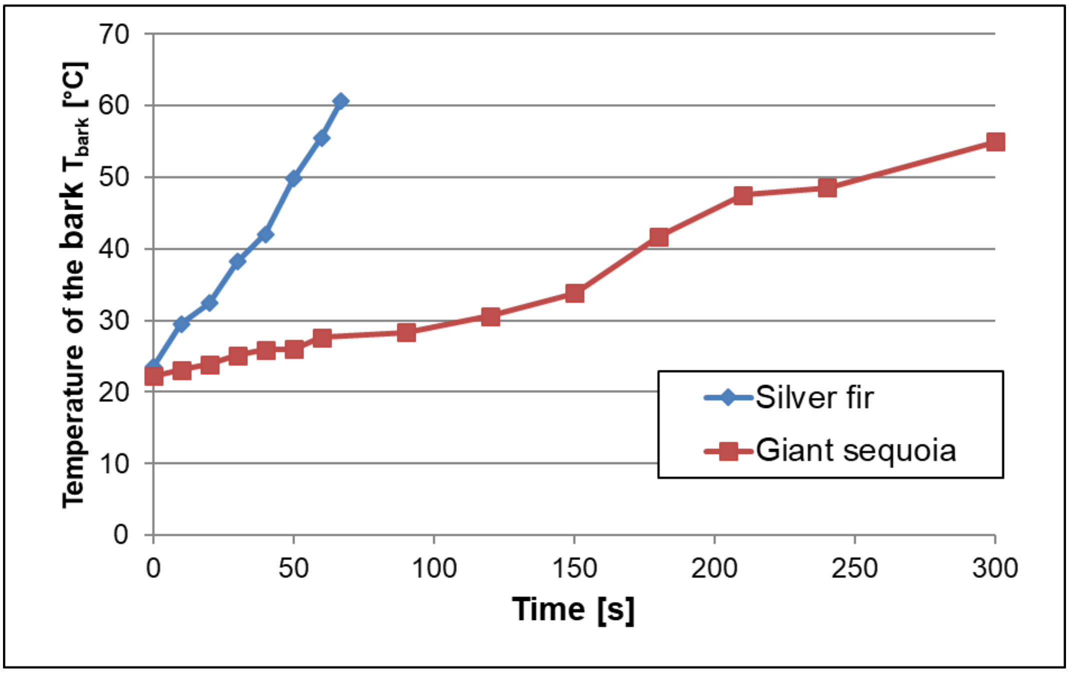

Figure S3: Diagram showing the temperature at the inner side of the bark as a function of time for bark samples stored at 0% relative humidity.

Discussion of the bark temperature-time curves (Figure S3)

The temperature-time curves show that bark samples with high fire tolerance can be distinguished from those with low fire tolerance. For all tree species whose bark samples have a value of less than 300 seconds, the vascular cambium would have been damaged, and the tree would not have survived a surface fire. Trees with high fire tolerance have values of 300 seconds and more.
The bark sample of the giant sequoia did not reach the critical temperature of 60°C at its inner side even after 300 seconds. Thus, the giant sequoia would have easily survived an average surface fire.
The bark of the silver fir, on the other hand, reached the critical temperature of 60°C at its inner side after only 67 seconds. This tree would not have survived a surface fire because the living cells of the vascular cambium would have been damaged.

Task 8: Calculate the mean sample thickness for each sample. Plot the time [s] to critical temperature 60°C (τ_{60}) on the y-axis as a function of the mean sample thickness [cm] for each tree species. You can use a spreadsheet program to calculate the mean and create the diagram. To determine the quantitative relationship between sample thickness and τ_{60}, you can include a trend line with a coefficient of determination. Discuss the results.

Tree species	Sample thickness [cm]	Critical temperature τ60 [s]
European beech	0.111	11.44
Silver fir	0.209	20.44
Scots pine	0.544	90.81
European larch	0.722	173.18
Cork oak	0.998	271.56
Giant sequoia	1.532	432.44

Fire tolerance of tree trunks

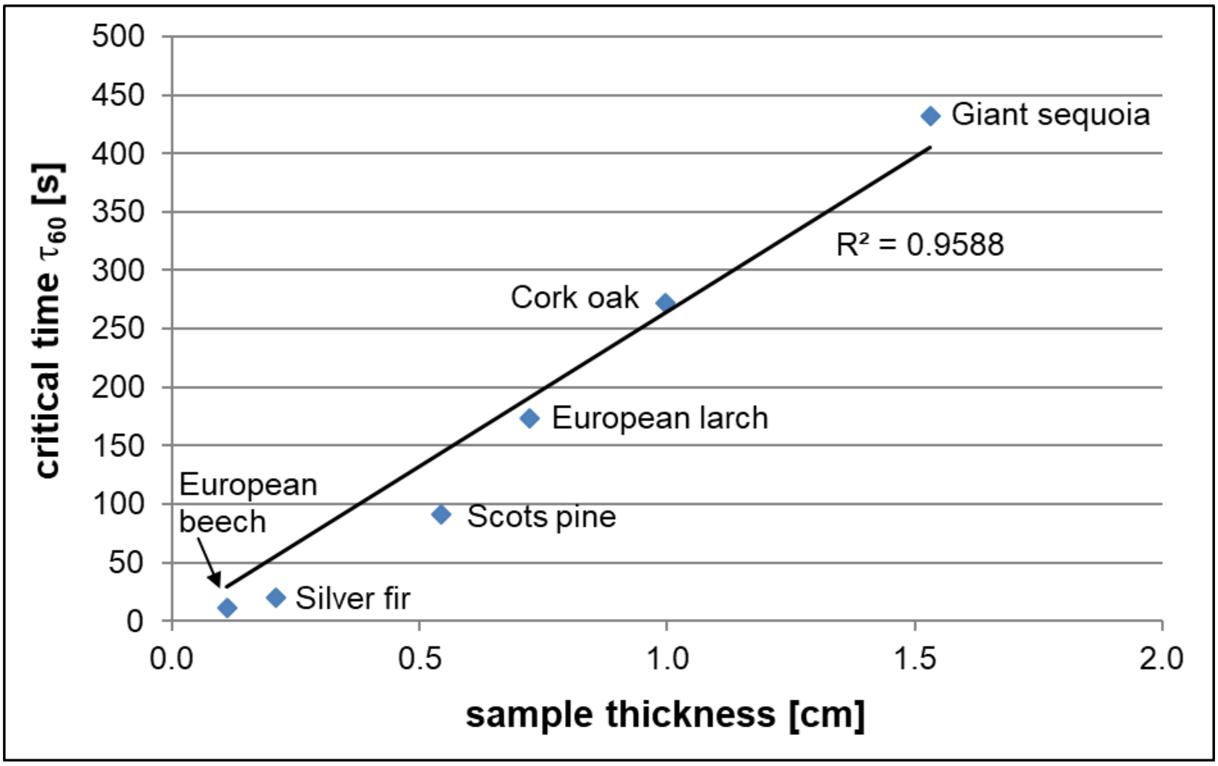

Figure S4: Diagram showing the critical temperature of the vascular cambium as a function of sample thickness for bark samples stored at 0% relative humidity.

Discussion of the critical temperature-sample thickness diagram (Diagram S4)

The graph shows the critical temperature of 60°C (τ_{60}) as a function of the bark thickness for various tree species. The trend line has a regression coefficient of 0.9588, indicating a linear relationship between the critical temperature τ_{60} at the vascular cambium and the bark thickness. We can therefore assume that the fire tolerance of a tree species depends on its ability to build up a thick bark layer as quickly as possible.
