# Supplementary material for: Is a Forest Fire a Natural Disaster? Investigating the Fire Tolerance of Various Tree Species—An Educational Module
Source: Biomimetics (Basel). 2024 Feb 15;9(2):114. doi: 10.3390/biomimetics9020114 (PMC10887004; doi:10.3390/biomimetics9020114)
Supplement: Supplementary file 1 [file biomimetics-09-00114-s001.zip › S2_fire-tolerance.pdf]

Total Wildfires and burned Acres in North-America incl. Alaska and Hawaii

The National Interagency Coordination Center at NIFC compiles annual wildland fire statistics for federal and state agencies. This information is provided through Situation Reports, which have been in use for several decades.
Prior to 1983, the federal wildland fire agencies did not track official wildfire data using current reporting processes. As a result, there is no official data prior to 1983 posted on this site.
Source: National Interagency Coordination Center
https://www.nifc.gov/sites/default/files/NICC/2-Predictive%20Services/Intelligence/Annual%20Reports/2022/annual_report.2.pdf

Year	Fires	km²	Acres
2022	68,988	30,351	7,500,000
2021	58,985	28,836	7,125,643
2020	58,950	40,964	10,122,336
2019	50,477	18,876	4,664,364
2018	58,083	35,481	8,767,492
2017	71,499	40,574	10,026,086
2016	67,743	22,298	5,509,995
2015	68,151	40,975	10,125,149
2014	63,312	14,551	3,595,613
2013	47,579	17,481	4,319,546
2012	67,774	37,742	9,326,238
2011	74,126	35,254	8,711,367
2010	71,971	13,851	3,422,724
2009	78,792	23,965	5,921,786
2008	78,979	21,418	5,292,468
2007	85,705	37,749	9,328,045
2006	96,385	39,958	9,873,745
2005	66,753	35,165	8,689,389
2004	65,461	32,771	8,097,880
2003	63,629	16,029	3,960,842
2002	73,457	29,076	7,184,712
2001	84,079	14,451	3,570,911
2000	92,250	29,920	7,393,493
1999	92,487	22,768	5,626,093
1998	81,043	5,381	1,329,704
1997	66,196	11,562	2,856,959
1996	96,363	24,548	6,065,998
1995	82,234	7,448	1,840,546
1994	79,107	16,485	4,073,579
1993	58,810	7,683	1,898,574
1992	87,394	8,377	2,069,929
1991	75,754	11,953	2,953,578
1990	66,481	18,703	4,621,621
1989	48,949	7,395	1,827,310
1988	72,750	20,272	5,009,290
1987	71,300	9,904	2,447,296
1986	85,907	11,004	2,719,162
1985	82,591	11,720	2,896,147
1984	20,493	4,647	1,148,409
1983	18,229	5,357	1,323,666

human-caused fires

Year	Fires	km²	Acres	% Fires	% km²
2022	61,429	13,639	3,370,169	89	45
2021	52,641	12,237	3,023,759	89	42
2020	53,563	24,276	5,998,813	91	59
2019	44,115	4,926	1,217,324	87	26
2018	51,576	22,826	5,640,489	89	64
2017	63,546	19,548	4,830,476	89	48
2016	60,932	15,243	3,766,610	90	68
2015	58,916	8,144	2,012,461	86	20
2014	55,679	6,405	1,582,770	88	44
2013	38,349	5,107	1,261,980	81	29
2012	58,331	10,118	2,500,249	86	27
2011	63,877	21,678	5,356,771	86	61
2010	64,807	5,275	1,303,449	90	38
2009	69,650	8,388	2,072,746	88	35
2008	70,093	13,881	3,429,991	89	65
2007	73,446	15,295	3,779,360	86	41
2006	80,220	17,826	4,404,844	83	45
2005	58,430	6,157	1,521,327	88	18
2004	54,101	3,904	964,800	83	12
2003	50,815	7,779	1,922,249	80	49
2002	62,022	12,453	3,077,119	84	43
2001	70,066	7,077	1,748,661	83	49

lightning-caused fires

Year	Fires	km²	Acres	% Fires	% km²
2022	7,467	17,025	4,206,960	11	56
2021	6,344	16,600	4,101,884	11	58
2020	5,387	16,687	4,123,523	9	41
2019	6,362	13,950	3,447,038	13	74
2018	6,507	12,655	3,127,003	11	36
2017	7,953	21,026	5,195,610	11	52
2016	6,790	7,055	1,743,385	10	32
2015	9,235	32,831	8,112,688	14	80
2014	7,933	8,146	2,012,843	13	56
2013	9,230	12,374	3,057,566	19	71
2012	9,443	27,624	6,825,989	14	73
2011	10,249	13,576	3,354,596	14	39
2010	7,164	8,576	2,119,275	10	62
2009	9,142	15,577	3,849,040	12	65
2008	8,856	7,537	1,862,477	11	35
2007	12,261	23,790	5,878,691	14	63
2006	16,165	22,132	5,468,901	17	55
2005	8,323	29,008	7,168,062	12	82
2004	11,384	28,373	7,011,023	17	87
2003	12,776	8,249	2,038,443	20	51
2002	11,435	16,582	4,097,593	16	57
2001	14,094	7,376	1,822,600	17	51

Europa https://joint-research-centre.ec.europa.eu/system/files/2022-10/Forest_Fires_Annual_report_2021_online.pdf

Annex Table 101 und 102

EUMED 5 = Portugal, Spain, Southern France, Italy, Greece

Year	Fires (number)	burnt area (ha)
**2022**		
**2021**	28,944	494,082
**2020**	30,661	215,126
**2019**	32,158	194,710
**2018**	26,434	109,808
**2017**	48,140	919,913
**2016**	35,801	341,760
**2015**	41,963	230,167
**2014**	25,746	139,005
**2013**	39,776	298,733
**2012**	56,593	543,181
**2011**	60,280	292,181
**2010**	47,632	262,545
**2009**	56,799	337,953
**2008**	41,389	171,746
**2007**	52,058	584,571
**2006**	52,263	299,622
**2005**	81,374	611,562
**2004**	61,176	369,717
**2003**	64,875	788,522
**2002**	58,761	315,277
**2001**	62,440	326,007
**2000**	74,006	631,950
**1999**	57,092	248,142
**1998**	74,793	559,748
**1997**	67,707	314,222
**1996**	62,399	243,379
**1995**	75,322	407,319
**1994**	57,215	734,195
**1993**	51,942	413,726
**1992**	52,134	355,983
**1991**	44,569	565,840
**1990**	45,338	646,822
**1989**	60,423	766,020
**1988**	33,701	463,775
**1987**	32,665	404,051
**1986**	27,439	517,203
**1985**	47,034	984,188
**1984**	29,997	353,958
**1983**	22,913	441,931
**1982**	26,081	405,432
**1981**	38,443	727,064
**1980**	27,749	506,328

Year	Fires (number)	km²	burnt area (ha)
2022			
2021	28,944	4,941	494,082
2020	30,661	2,151	215,126
2019	32,158	1,947	194,710
2018	26,434	1,098	109,808
2017	48,140	9,199	919,913
2016	35,801	3,418	341,760
2015	41,963	2,302	230,167
2014	25,746	1,390	139,005
2013	39,776	2,987	298,733
2012	56,593	5,432	543,181
2011	60,280	2,922	292,181
2010	47,632	2,625	262,545
2009	56,799	3,380	337,953
2008	41,389	1,717	171,746
2007	52,058	5,846	584,571
2006	52,263	2,996	299,622
2005	81,374	6,116	611,562
2004	61,176	3,697	369,717
2003	64,875	7,885	788,522
2002	58,761	3,153	315,277
2001	62,440	3,260	326,007
2000	74,006	6,320	631,950
1999	57,092	2,481	248,142
1998	74,793	5,597	559,748
1997	67,707	3,142	314,222
1996	62,399	2,434	243,379
1995	75,322	4,073	407,319
1994	57,215	7,342	734,195
1993	51,942	4,137	413,726
1992	52,134	3,560	355,983
1991	44,569	5,658	565,840
1990	45,338	6,468	646,822
1989	60,423	7,660	766,020
1988	33,701	4,638	463,775
1987	32,665	4,041	404,051
1986	27,439	5,172	517,203
1985	47,034	9,842	984,188
1984	29,997	3,540	353,958
1983	22,913	4,419	441,931
1982	26,081	4,054	405,432
1981	38,443	7,271	727,064
1980	27,749	5,063	506,328
